# Internal Limiting Membrane Peeling for Persistent Submacular Fluid after Successful Repair of Diabetic Tractional Retinal Detachment

**DOI:** 10.1155/2019/8074960

**Published:** 2019-10-23

**Authors:** Byung Ju Jung, Sohee Jeon, Kook Lee, Jiwon Baek, Won Ki Lee

**Affiliations:** ^1^Apgujung St. Mary's Eye Center, Seoul, Republic of Korea; ^2^Keye Eye Center, Seoul, Republic of Korea; ^3^Department of Ophthalmology, Seoul St. Mary's Hospital, College of Medicine, The Catholic University of Korea, Seoul, Republic of Korea; ^4^Department of Ophthalmology, Bucheon St. Mary's Hospital, College of Medicine, The Catholic University of Korea, Seoul, Republic of Korea; ^5^Department of Ophthalmology, Nune Eye Hospital, Seoul, Republic of Korea

## Abstract

This study is for reporting the outcomes of internal limiting membrane (ILM) peeling on persistent submacular fluid (PSF) after otherwise successful pars plana vitrectomy (PPV) for diabetic tractional retinal detachment (TRD). In this retrospective case series, five consecutive patients (5 eyes) who exhibited PSF following successful repair of diabetic TRD were included. The second operation was performed to remove ILM. The area of ILM peeling was expanded up to the major vascular arcade. Only air tamponade was used. The median interval between the first PPV and the second PPV with ILM peeling was 4.8 months (range: 4–6 months). PSF resolved completely within one (2 eyes) or 2 months after ILM peeling. The median logMAR best-corrected visual acuity (BCVA) was improved from 1.00 (Snellen equivalent 20/200) to 0.70 (Snellen equivalent 20/100). In conclusion, wide ILM peeling is an effective treatment option for PSF subsequent to successful repair of diabetic TRD. ILM peeling might increase the elasticity of retina, thereby allowing the retina to flatten. This procedure can induce faster retinal reattachment in diabetic TRD involving the macula.

## 1. Introduction

Tractional retinal detachment (TRD) that involves the macula is the main cause of permanent vision loss in patients with diabetic retinopathy and requires prompt surgical intervention [1, 2]. With the small-gauge vitrectomy system, anatomical success rate after pars plana vitrectomy (PPV) is reported over 90% [3, 4]. However, persistent submacular fluid (PSF) is sometimes observed postoperatively in patients with diabetic TRD involving or threatening the fovea, despite complete removal of the epiretinal fibrovascular membranes. PSF can be detected by optical coherence tomography (OCT) after absorption of tamponade gas or immediately after operation in cases with silicone tamponade or not requiring tamponade. In the case series of Barzideh and Johnson, PSF took 6–13 months to resolve and accounted for delayed visual recovery [5]. They found no obvious vascular leakage by fluorescein angiography, suggesting that the PSF might represent residual, viscous subretinal fluid secondary to the original TRD. In a recent prospective study including 23 patients (24 eyes) with macula-off diabetic TRD who underwent successful PPV, the prevalence of PSF on spectral-domain OCT (SD-OCT) was 100% at 2 months, 91.7% at 3 months, 70.8% at 6 months, 25.0% at 9 months, and 4.2% at 1 year [6]. It means that delayed residual subfoveal fluid resorption is a common phenomenon in clinically successful surgery. Although most cases resolve with observation, long-standing submacular fluid can damage photoreceptors, cause permanent visual loss, trigger retinal thinning, and possibly contribute to macular hole formation [7, 8].

In eyes with myopic traction maculopathy, the outer retina is stretched along with the posterior staphyloma, but the inner retina is not well-elongated, causing schisis-like deformation. Tractional force imparted by the rigid internal limiting membrane (ILM) and/or residual premacular vitreous cortex has been suggested to be responsible for the inability of the inner retina to conform to the posterior staphyloma [9–12]. Vitrectomy with ILM peeling has become a widely accepted treatment option for this disease entity.

We speculate that stiffness of the contracted retina induced by the tractional membrane would not fully recover after removal of the fibrovascular membrane. The detached retina in the macular area might permit residual fluid to ingress and persist in the subretinal space. We hypothesized that removal of the rigid and contracted ILM over the detached retina would restore the elasticity of the retina and facilitate reattachment of the detached retina. In five consecutive patients who exhibited PSF after successful repair of diabetic TRD, ILM peeling was performed as a secondary procedure. In this report, we describe the efficacy of this surgical approach.

## 2. Materials and Methods

This study was a retrospective case series and was approved by the Institutional Review Board of Seoul St. Mary's Hospital, Seoul, South Korea, and followed all relevant tenets of the Declaration of Helsinki.

We reviewed the charts and imaging records of consecutive patients who exhibited prolonged PSF after successful PPV to treat diabetic TRD and underwent secondary operation of ILM peeling. Patients who had fibrovascular membranes not completely removed in the first operation, or who developed a new epiretinal membrane on SD-OCT, or who exhibited rhegmatogenous retinal detachment (RRD) were excluded. All the surgeries in this study were performed by a single vitreoretinal surgeon (WKL) at Seoul St. Mary's Hospital, the Catholic University of Korea, between January 2015 and October 2017.

All patients underwent ophthalmologic examinations, including assessment of best-corrected visual acuity (BCVA) and refraction; slit-lamp biomicroscopy; fundoscopy with dilated pupils; fundus photography (VX-20 fundus camera; Kowa, Tokyo, Japan) or ultra-widefield fundus photography (Optos, Dunfermline, UK); and SD-OCT (Spectralis, Heidelberg Engineering, Heidelberg, Germany). In the first operation, PPV using a Constellation 23- gauge (G) vitrectomy instrument (Alcon Laboratories, Inc., Fort Worth, TX, USA) was performed for the repair of diabetic TRD involving the macula. Intravitreal antivascular endothelial growth factor (anti-VEGF) was preoperatively injected in all patients. The surgeon removed all visible tractional fibrovascular membranes using vitreous cutter, horizontal scissors, and forceps. When necessary, one 20-G sclerotomy port was created, and 20-G curved microscissors were used for the delamination procedure. Perfluorocarbon liquid was used to drain internal subretinal fluid when retinal breaks were detected. Endolaser photocoagulation was applied to retinal breaks and peripheral retina. The surgeon determined the necessity of tamponade at the end of surgery and chose an appropriate tamponade, such as octafluoropropane (C3F8) gas or silicone oil. Face-down positioning was recommended for at least 2 weeks in cases with gas or silicone tamponade.

Secondary PPV for ILM peeling was scheduled when no improvement in the amount of PSF on OCT was apparent during more than 3 months of follow-up after gas absorption. To remove the ILM, 0.5% indocyanine green (ICG) dye (Dongindang Inc., Seoul, Korea) was injected slowly onto the posterior pole with the fluid-filled state and ILM was stained for 60 seconds. After flushing the ICG dye, ILM was pinched and peeled using forceps (Grieshaber advanced DSP ILM forceps 723.44; Alcon Laboratories Inc, Fort Worth, Texas, USA). ILM peeling included the area over the entire posterior pole and expanded up to the major vascular arcade and near to the optic disc. Fluid-air exchange was performed for all eyes, and face-down positioning was recommended for 1–3 days.

## 3. Results

Five patients (5 eyes) were eligible for inclusion. Their median age was 58 years (range: 41–59 years). Two patients were male and three were female. The tamponades in the first operation were air (*n* = 1), 14% C3F8 gas (*n* = 3), and silicone oil (*n* = 1). One or two months later, overall, the retina was well attached without any remaining membranes; however, PSF was evident on OCT. The median interval between primary PPV and secondary PPV with ILM peeling was 4 months (range: 4–6 months). The median logMAR BCVA before ILM peeling was 1.00 (Snellen equivalent, 20/200).

All cases exhibited complete resolution of PSF and retinal reattachment. The time to reattachment after ILM peeling was less than 2 months in all cases and less than one month in 3 eyes. The final median logMAR BCVA was 0.70 (Snellen equivalent 20/100). Clinical and demographic characteristics of all subjects are shown in Table 1.

### 3.1. Example Case 1

**Figure 1:**
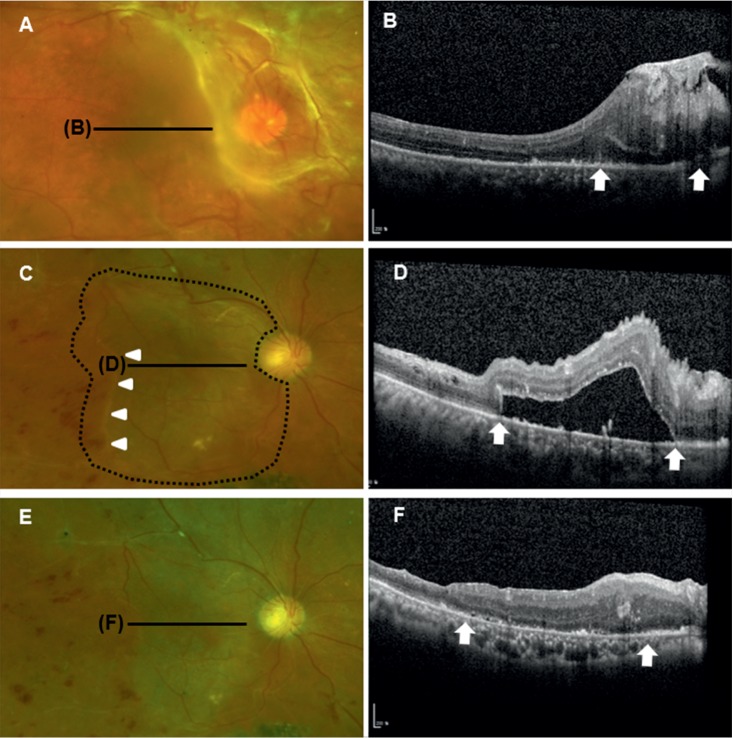
Multimodal imaging of a macula-threatening, diabetic, tractional retinal detachment in a 58-year-old male. (a) Preoperative fundus photograph showing extensive tractional membranes around the optic disc. (b) A spectral-domain optical coherence tomography (SD-OCT) scan through the fovea demonstrates the extent of retinal detachment (arrows). (c) and (d) Persistent subfoveal detachment at 6 months postoperatively (arrows and arrowheads). The extent of internal limiting membrane (ILM) peeling is marked (black dotted line). (e) and (f) Completely reattached retina at 1 month after secondary surgery with ILM peeling.

A 58-year-old male with newly diagnosed diabetes presented with a decreased visual acuity of both eyes for 3 months. He did not receive any ocular examination or treatment before. Initial logMAR BCVA was 1.70 (Snellen 20/1,000) in the right eye and 0.60 (Snellen 20/80) in the left eye. Fundus examination revealed severe fibrovascular proliferation around the optic disc and superior temporal vascular arcade of right eye (Figure 1(a)). The fovea was dragged nasally due to tractional membranes and detached on SD-OCT (Figure 1(b)). Proliferative diabetic retinopathy with retinal neovascularization was also shown in the left eye, but operative approach was not required. Panretinal photocoagulation was performed prior to the PPV of right eye, and anti-VEGF (bevacizumab) was injected 3 days before the surgery. During PPV, epiretinal fibrovascular membranes were removed completely, and a small retinal tear was noted at 2 o'clock in the midperipheral retina. Endolaser photocoagulation and 14% C3F8 gas tamponade were performed. At 6 weeks after the operation, visual acuity was logMAR 1.70 (Snellen 20/1,000). Although the retina was well attached, persistent subretinal fluid was detected in the posterior pole on OCT. The amount of collected fluids was increased postoperatively. During 6 months (the next 4.5 months of F/U), the amount of remains unchanged and visual acuity did not improve.

On both fundus examination and fundus photography, there was no obviously visible tractional or epiretinal membrane. Focal and thin epiretinal membrane was shown at the edge of the optic disc on OCT (Figure 1(d)). It was thought not to be associated with tractional cause. However, the presence of a tractional force was suspected, given the severely distorted superior retinal vasculature and significant dragging of the outer retinal tissue at the margin of the PSF (Figures 1(c) and 1(d)). We speculated that the rigid ILM imparted a tractional force hindering morphologic restoration. Wide ILM peeling (including over the superior and inferior retinal vasculature, for a one-disc-diameter distance from the PSF margin) was performed. Fluid-air exchange was performed, and the patient was instructed to maintain a face-down position for 3 days. Retinal reattachment was rapid, commencing 1 week after the second operation. After 1 month, the retina was completely reattached, and the previously distorted retinal vasculature was significantly straightened (Figures 1(e) and 1(f)). The final logMAR BCVA improved to logMAR 1.0 (Snellen 20/200) at 1 month after ILM peeling and maintained during 12 months.

### 3.2. Example Case 2

**Figure 2:**
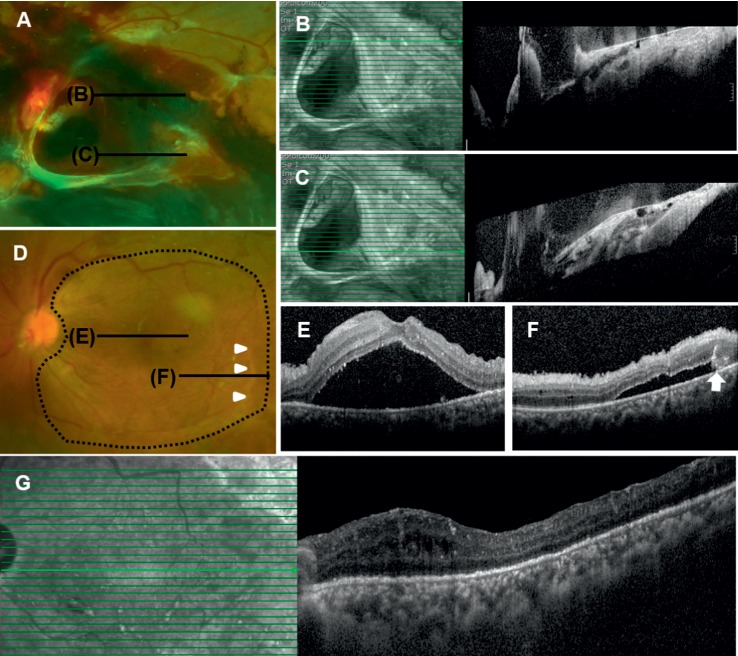
A 41-year-old female with tractional retinal detachment (TRD) involving the fovea of the left eye. (a),(b), and (c) Preoperative fundus photograph and SD-OCT images. (d), (e), and (f) Persistent submacular fluid at 4 months postoperatively (arrows and arrowheads). Extensive ILM peeling was performed (black dotted line). (g) At 1 month after wide ILM peeling, the retina was completely reattached.

A 41-year-old female presented with decreased visual acuity of left eye. She was newly diagnosed with diabetes and has no history of ophthalmologic treatment. Initial BCVA was hand motion in the left eye and logMAR 0.1 (Snellen 0.8) in the right eye. Fundus examination revealed proliferative diabetic retinopathy with retinal neovascularization of right eye and TRD involving the fovea with a subhyaloid hemorrhage in the left eye. Diffuse tractional membranes extended along the major vascular arcades (Figure 2(a)), and extensive subretinal fluid and a subhyaloid hemorrhage were detected on the macula on SD-OCT examination (Figures 2(b) and 2(c)). The patient underwent a planned PPV as described for Example 1. All tractional membranes were removed using a vitreous cutter, disposable ILM forceps, and microscissors; the surgeon confirmed that no definite retinal break was apparent. After panretinal endolaser photocoagulation, C3F8 was injected as tamponade because of the possibility of missed tiny breaks. During 4 months of postoperative follow-up, the PSF did not decrease on SD-OCT, and the logMAR BCVA remained at 1.40 (Snellen 20/500) (Figures 2(d) and 2(e)). Extensive ILM peeling including over the entire posterior pole was performed to cover the detached retina (Figure 2(d)). No epiretinal membrane was detected on the detached macula during surgery. Perfluorocarbon application was used to rule out RRD; no additional retinal break was evident. Fluid-air exchange was performed without any tamponade, and the patient was instructed to remain in the face-down position for 3 days. At the 1-month follow-up, the fovea was totally reattached on SD-OCT (Figure 2(f)) and the final logMAR BCVA improved to 0.70 (Snellen 20/100) at 1 month and maintained until 18 months postoperatively.

## 4. Discussion

Persistent submacular fluid has also been reported after uneventful scleral buckling or PPV to treat RRD [8, 13]. An excessive deposit of proteins in the subretinal fluid due to a long-standing detachment might contribute to the persistence of fluid. The presence of proteins would make the reabsorbance extremely difficult. While most will resolve with observation, long-standing submacular fluid can cause damage to photoreceptor and permanent visual loss. Itakura and Kishi proposed pneumatic displacement for this condition under the assumption that displaced subretinal fluid might be absorbed more robustly by the peripheral retinal pigment epithelium [14]. Reichstein et al. introduced a novel technique, PPV with subretinal injection of balanced salt solution and gas instillation, which was designed to address the proteinaceous nature of the fluid by diluting the fluid as well as displacing it with intraocular gas [7]. However, to the best of our knowledge, no study has yet described how to resolve PSF after diabetic TRD. In our series, PSF resolved completely within 1 or 2 months after secondary operation featuring extensive ILM peeling, and visual acuity improved. The PSF had remained unchanged for 4–6 months after the first operations. At the time of the first PPV, four of five eyes received C3F8 gas (three eyes) or silicone (one eye) tamponade, and prone positioning was maintained for at least 2 weeks. At the second PPV with ILM peeling, only fluid-air exchange was performed and prone positioning was maintained for only 3 days. Therefore, pneumatic displacement effect proposed for PSF in RRD cases seems not to be an appropriate explanation for the fluid resolution in our cases. We postulate that structural macular detachment is the primary event, which is attributable to the decreased elasticity or shortening of the retina in long-standing diabetic TRD. Fluids might ingress and persist beneath the retina not restoring its contour and being lifted. In our series, the areas of PSF corresponded to the detached area noted before operation. Subretinal fluid is rather increased postoperatively as shown in case 1 (Figure 1). It is possible that the subretinal fluids in the periphery may move to the macular area. ILM peeling over the posterior pole might increase the elasticity of retina, thereby allowing the retina to flatten.

It is commonly seen that a detached retina associated with severe fibrovascular proliferation cannot be completely attached on the operation table even though all fibrovascular tissues are successfully removed. Endolaser cannot be effectively delivered to such areas. In instances of iatrogenic or preexisting retinal break, the retina cannot be completely approximated to the retinal pigment epithelium despite repeated subretinal fluid drainage. And laser burns around the retinal tears are not made enough without scleral indentation. The thin peripheral retina seems to restore its elasticity rapidly and to become attached, and laser burns are readily made using a table laser within 1-2 weeks after operation under gas or a silicone tamponade. However, a longer interval might be required for restoration of the configuration and elasticity of the thick macular retina.

Based upon the promising results in the current study, ILM peeling can be considered as a primary procedure to induce faster macula reattachment in diabetic TRD involving the macula. The ILM is a fine multilaminar membrane composed of the expanded footplates of Müller glial cells. The thickness of ILMs ranges from 1 to 2 *μ*m. Under physiological conditions, the hyaloid membrane adheres to the ILM. Under pathologic conditions such as diabetic retinopathy, migrated fibroblasts and fibrinous materials attach to the surface of the ILM, increasing the ILM thickness. Contraction of these materials within the ILM may impart tangential traction to the underlying retina, although no fibrovascular membrane may be observed above the ILM [15]. Moreover, ILM works as a framework for proliferative process with myofibroblast originated from cortical vitreous remnants and residual blood [16, 17]. Some studies had reported the incidence of postoperative macular pucker was over 10% in ILM nonpeeling cases [18, 19]. ILM removal is regarded very effective to remove posterior vitreous remnants and prevent postoperative ERM formation [20, 21]. ILM peeling in diabetic TRD also has a beneficial role in eliminating potential tractional sources as well as possibility of proliferative membrane formation.

Obviously, this study has a limitation stemming from its small sample size and lack of a control group. PSF might be resolved spontaneously, and it is very hard to recommend the second operation in real clinical practice. Although the number is small, our cases revealed dramatic resolution of PSF with visual improvement. It is worthwhile to perform further comparative studies with more patients to confirm our observations.

## 5. Conclusions

Wide ILM peeling was an effective treatment option for persistent macular detachment subsequent to otherwise successful repair of diabetic TRD. This procedure may increase the elasticity of a retina that has been detached and shortened by traction over a long period of time, facilitating retinal reattachment. It may be worthwhile to perform ILM peeling as a primary procedure in eyes with severe diabetic TRD although no obvious epiretinal traction is apparent.

## Figures and Tables

**Table 1:** Clinical characteristics of 5 eyes with ILM peeling in diabetic tractional retinal detachment.

Case no.	Sex/age	Eye	Tamponade in the first operation	Interval to ILM peeling (m)	Time to reattachment after ILM peeling (m)	LogMAR BCVA (Snellen equivalent)		
						Before the first operation	Last F/U before ILM peeling	Last F/U after ILM peeling
1	M/58	L	C3F8	6	<1	1.70 (20/1000)	1.70 (20/1000)	1.0 (20/200)
2	F/41	L	C3F8	4	<1	2.28 (HM)	1.40 (20/500)	0.70 (20/100)
3	M/59	L	C3F8	4	2	1.00 (20/200)	1.00 (20/200)	0.80 (20/125)
4	F/48	R	Air	4	1	0.40 (20/50)	0.40 (20/50)	0.20 (20/32)
5	F/58	R	Silicone oil	6	2	0.80 (20/125)	0.70 (20/100)	0.50 (20/63)

M, male; F, female; R, right; L, left; m, month; BCVA, best-corrected visual acuity; C3F8, octafluoropropane; F/U, follow-up; ILM, internal limiting membrane; HM, hand motion.

## Data Availability

The data used to support the findings of this study are available from the corresponding author upon request.

## References

[1] Rice T. A., Michels R. G., Rice E. F. (1983). Vitrectomy for diabetic traction retinal detachment involving the macula. *American Journal of Ophthalmology*.

[2] Fong D. S., Ferris F. L., Davis M. D., Chew E. Y. (1999). Causes of severe visual loss in the early treatment diabetic retinopathy study: ETDRS report no. 24. *American Journal of Ophthalmology*.

[3] Mikhail M., Ali-Ridha A., Chorfi S., Kapusta M. A. (2017). Long-term outcomes of sutureless 25-G+ pars-plana vitrectomy for the management of diabetic tractional retinal detachment. *Graefe’s Archive for Clinical and Experimental Ophthalmology*.

[4] Storey P. P., Ter-Zakarian A., Philander S. A. (2017). Visual and anatomical outcomes after diabetic traction and traction-rhegmatogenous retinal detachment repair. *Retina*.

[5] Barzideh N., Johnson T. M. (2007). Subfoveal fluid resolves slowly after pars plana vitrectomy for tractional retinal detachment secondary to proliferative diabetic retinopathy. *Retina*.

[6] Karimov M. I., Gasymov E. M., Aliyeva I. J., Akhundova L. A., Rustambayova G. R., Aliyev K. D. (2018). An optical coherence tomography study of residual subfoveal fluid after successful pars plana vitrectomy in patients with diabetic tractional macular detachment. *Eye*.

[7] Reichstein D. A., Larsen B. P., Kim J. E. (2013). Management of persistent subretinal fluid following retinal detachment repair. *JAMA Ophthalmology*.

[8] Tee J. J. L., Veckeneer M., Laidlaw D. A. (2016). Persistent subfoveolar fluid following retinal detachment surgery: an SD-OCT guided study on the incidence, aetiological associations, and natural history. *Eye*.

[9] Bando H., Ikuno Y., Choi J.-S., Tano Y., Yamanaka I., Ishibashi T. (2005). Ultrastructure of internal limiting membrane in myopic foveoschisis. *American Journal of Ophthalmology*.

[10] Fujimoto M., Hangai M., Suda K., Yoshimura N. (2010). Features associated with foveal retinal detachment in myopic macular retinoschisis. *American Journal of Ophthalmology*.

[11] Hayashi W., Shimada N., Hayashi K. (2011). Retinal vessels and high myopia. *Ophthalmology*.

[12] Kuhn F. (2003). Internal limiting membrane removal for macular detachment in highly myopic eyes. *American Journal of Ophthalmology*.

[13] Wolfensberger T. J., Gonvers M. (2002). Optical coherence tomography in the evaluation of incomplete visual acuity recovery after macula-off retinal detachments. *Graefe’s Archive for Clinical and Experimental Ophthalmology*.

[14] Itakura H., Kishi S. (2009). Intravitreal injection of 0.3 ml of SF6 gas for persistent subfoveal fluid after scleral buckling for rhegmatogenous retinal detachment. *Graefe’s Archive for Clinical and Experimental Ophthalmology*.

[15] Eckardt C., Eckardt U., Groos S., Luciano L., Reale E. (1997). Entfernung der membrana limitans interna bei makulalöchern. *Der Ophthalmologe*.

[16] Kampik A., Green W. R., Michels R. G., Nase P. K. (1980). Ultrastructural features of progressive idiopathic epiretinal membrane removed by vitreous surgery. *American Journal of Ophthalmology*.

[17] Michalewska Z., Bednarski M., Michalewski J., Jerzy N. (2013). The role of ILM peeling in vitreous surgery for proliferative diabetic retinopathy complications. *Ophthalmic Surgery, Lasers and Imaging Retina*.

[18] Lewis H., Abrams G. W., Blumenkranz M. S., Campo R. V. (1992). Vitrectomy for diabetic macular traction and edema associated with posterior hyaloidal traction. *Ophthalmology*.

[19] Tachi N., Ogino N. (1996). Vitrectomy for diffuse macular edema in cases of diabetic retinopathy. *American Journal of Ophthalmology*.

[20] Gandorfer A., Messmer E. M., Ulbig M. W., Kampik A. (2000). Resolution of diabetic macular edema after surgical removal of the posterior hyaloid and the inner limiting membrane. *Retina*.

[21] Yamamoto T., Hitani K., Sato Y., Yamashita H., Takeuchi S. (2005). Vitrectomy for diabetic macular edema with and without internal limiting membrane removal. *Ophthalmologica*.

